# Stopping criteria for ending autonomous, single detector radiological source searches

**DOI:** 10.1371/journal.pone.0253211

**Published:** 2021-06-17

**Authors:** Gregory R. Romanchek, Shiva Abbaszadeh

**Affiliations:** 1 Department of Nuclear, Plasma, Radiological Engineering, Grainger College of Engineering, University of Illinois at Urbana-Champaign, Urbana, Illinois, United States of America; 2 Department of Electrical and Computer Engineering, Jack Baskin School of Engineering, University of California Santa Cruz, Santa Cruz, California, United States of America; Fuzhou University, CHINA

## Abstract

While the localization of radiological sources has traditionally been handled with statistical algorithms, such a task can be augmented with advanced machine learning methodologies. The combination of deep and reinforcement learning has provided learning-based navigation to autonomous, single-detector, mobile systems. However, these approaches lacked the capacity to terminate a surveying/search task without outside influence of an operator or perfect knowledge of source location (defeating the purpose of such a system). Two stopping criteria are investigated in this work for a machine learning navigated system: one based upon Bayesian and maximum likelihood estimation (MLE) strategies commonly used in source localization, and a second providing the navigational machine learning network with a “stop search” action. A convolutional neural network was trained via reinforcement learning in a 10 m × 10 m simulated environment to navigate a randomly placed detector-agent to a randomly placed source of varied strength (stopping with perfect knowledge during training). The network agent could move in one of four directions (up, down, left, right) after taking a 1 s count measurement at the current location. During testing, the stopping criteria for this navigational algorithm was based upon a Bayesian likelihood estimation technique of source presence, updating this likelihood after each step, and terminating once the confidence of the source being in a single location exceeded 0.9. A second network was trained and tested with similar architecture as the previous but which contained a fifth action: for self-stopping. The accuracy and speed of localization with set detector and source initializations were compared over 50 trials of MLE-Bayesian approach and 1000 trials of the CNN with self-stopping. The statistical stopping condition yielded a median localization error of ~1.41 m and median localization speed of 12 steps. The machine learning stopping condition yielded a median localization error of 0 m and median localization speed of 17 steps. This work demonstrated two stopping criteria available to a machine learning guided, source localization system.

## Introduction

Radiological source surveying and localization protocols are a necessary component in the defense against rogue nuclear sources. While the mapping and pinpointing of radioactive sources was originally tasked to human surveyors with handheld detectors, these tasks can now be accomplished with autonomous robotic vehicles hoisting detectors–unmanned aerial or ground vehicles (UAVs, UGVs), for example. These autonomous, single-detector systems can leverage the benefits of machine learning (ML) algorithms for navigation and a wealth of statistical algorithms for estimating source location. Maximum likelihood estimation (MLE) and Bayesian approaches are popular statistical techniques for source localization in detector-networks [1–5] and single detector systems [6–8] alike. Further, these statistical techniques can be easily modified to work in unison with ML-navigation. However, doing so leaves the system with a fundamental, unmet requirement: a stopping criterion–a rule for when the autonomous system can end its search and yield the estimated source location. This work investigates using a statistical stopping criterion for a joint ML-statistical system and compares the performance with a purely ML approach.

MLE and Bayesian approaches are well-suited to capture the inherent randomness in radioactive decay and well-defined measurement statistics. However, these purely statistical methods compound in complexity when addressing the complicated features of source localization scenarios, such as attenuating barriers [2, 3], multiple sources [1, 4, 5, 9], or poor signal-to-noise ratio [10]. Much work is based upon a robust detector-network infrastructure [1–5]–a factor limiting the applicability of such approaches. Statistical algorithms developed for single detector systems either have no navigational control [6, 11], a predefined path [7–9, 12], or no stopping criteria [13]. An algorithm incorporating solutions to all the listed problems, while feasible, may be constrained by the required computation time, functionally eliminating any real-time analysis for navigational decisions. Due to these complexities, a combination of ML and statistics is a promising alternative to a purely statistical approach.

Within recent years, the combination of reinforcement learning (RL) with deep neural network architecture fuses navigation and localization strategies into a single algorithm [14, 15]. RL is a category of ML in which an agent responds to an environment with predefined actions to achieve a set goal. How well the agent performs is scored based on a reward function. In RL, the reward function is maximized to achieve an optimal action policy. In the context of source localization:

1. the agent is a detector-carrying entity;
2. the environment is the surveying area, relevant obstacles, background radiation, and source radiation;
3. the actions are discrete movements (e.g., up, down, left, right);
4. the goal is to navigate towards (localize) the radioactive source;
5. and the reward is based upon how quickly (in how many steps) the source is localized.

Q-learning and double Q-learning algorithms have been implemented as a training mechanism for RL in which a model of the environment is not needed for finding an optimal action policy [16]. This methodology has been successfully demonstrated in Zheng at al. [14], where an RL detector-agent could localize a source 44% faster than gradient search and uniform search methods.

Currently, RL localization strategies with UAVs terminate using perfect knowledge–they use the ground-truth source location (which would not be available in the field) to determine when to end a run [14, 15]. The effectiveness of the stopping criterion ultimately determines the accuracy of localization strategies. With current models, in the absence of true source location or human intervention, navigation would proceed indefinitely. This work investigates two types of stopping criteria for RL-guided source localization: 1) the first is based on popular MLE and Bayesian approaches for source localization, operating independently from RL-navigation, and 2) the second incorporates a stopping action into the RL-navigational algorithm itself, eliminating the need for statistical inference entirely.

## Materials and methods

In this section, we first define the simulated environment in which tests were carried out and in which the neural networks (one with a stopping action and the other without) were trained. Then, we provide an overview of the MLE-Bayesian scheme for source location estimation and search termination. The neural network architecture and training details are presented in the subsequent two subsections: first for the navigational network, and second for the navigational network with stopping.

### Simulation environment and data

**Fig 1:**
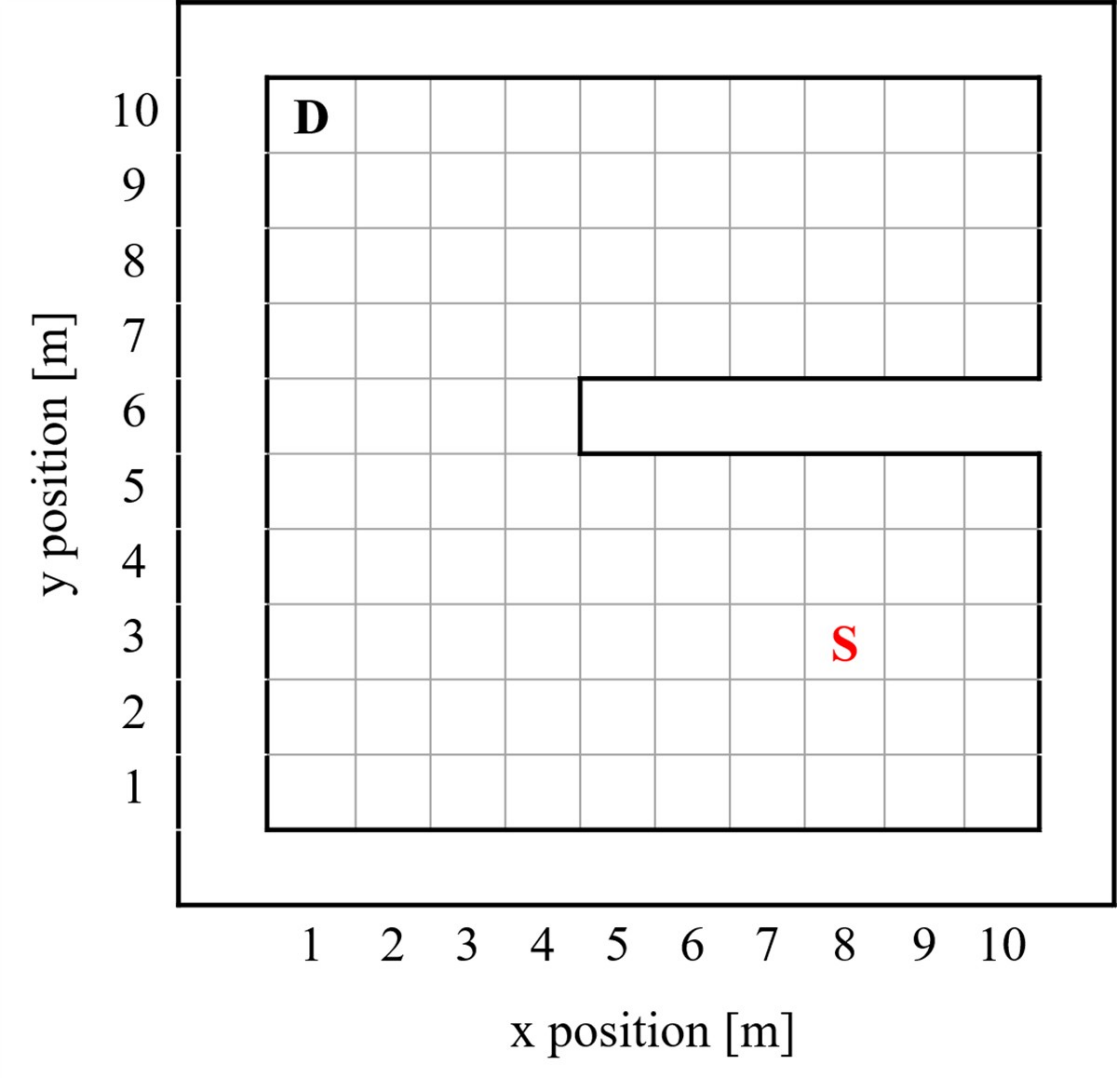
Simulated environment. Diagram of a possible configuration of the simulation environment with the detector-agent marked with the “D” and the source location marked with the “S”. A wall is seen protruding from the right side of the environment. Detector-agent location is restricted to the center of grid locations.

Tests and training were conducted in a simulated, instance-based environment with a 10 m × 10 m grid and 1 m grid spacing. Within this environment, a single wall extruding from a random position on the perimeter with variable length and occupying grid points may be present depending on the instance. A radioactive test source with intensity *I* cnts/s–defined such that a detector 1 m away would detect *I* counts in 1 s–is placed randomly in an open position. A background intensity of *b* cnts/s was set for all grid points–defined such that a detector on any grid point would detect an average of 25 counts in 1 s in the absence of source. This intensity was selected based upon the average background radiation levels on the University of Illinois at Urbana-Champaign (UIUC) campus with a Kromek D3S hand-held detector [17]. A diagram of an instance of this environment can be seen in Fig 1.

A model for radiation detection at each grid point was implemented based upon source strength, background strength, and radiation attenuation. The probability of observing *m* counts measured by a detector during a unit-time interval was modelled by a Poisson distribution:

$$P(m|\lambda )=\frac{e^{-\lambda }\lambda^{m}}{m!}$$ (1)

where *λ* is the total intensity of radiation present (including both source and background), and *m* is the number of counts detected. The total intensity *λ* is then:

$$\lambda \approx b+I\cdot d^{-2}\cdot 1_{\left\{\mathrm{not}\;\mathrm{blocked}\right\}}$$ (2)

Here, *d* is the Euclidean distance between the detector and the source. The unit function $1_{\left\{not\;blocked\right\}}$ equals one when the “not blocked” condition is met, otherwise it is zero. Using a simplified assumption of attenuation, if a wall is present in the simulated environment, it blocks all source counts from passing through it, preventing source signal at certain grid locations. Dividing the source intensity by the distance-squared approximately accounts for geometric efficiency and the detector solid angle. Thus, after a source with intensity *I* is placed, the expected intensity *λ* can be computed for each grid point by sampling from a Poisson distribution defined in Eq 1. We note this methodology assumes source gammas are fully attenuated by obstructions and that gammas do not scatter off of obstruction surfaces. These assumptions result in fewer counts than potentially expected in the vicinity near obstructions–either scattered back on the source-side or penetrated through on the shielded-side. The environment model used in this work can be expanded to include the physics of both these potentialities, but they are not expected to have a substantial effect on the RL performance, and the necessary material-modeling is beyond the scope of this implementation. Source intensity *I* differs between the training and evaluation steps, and so will be defined in the respective sections. A detector can occupy any grid point that is not occupied by a wall and collection times are limited to 1 s.

**Fig 2:**
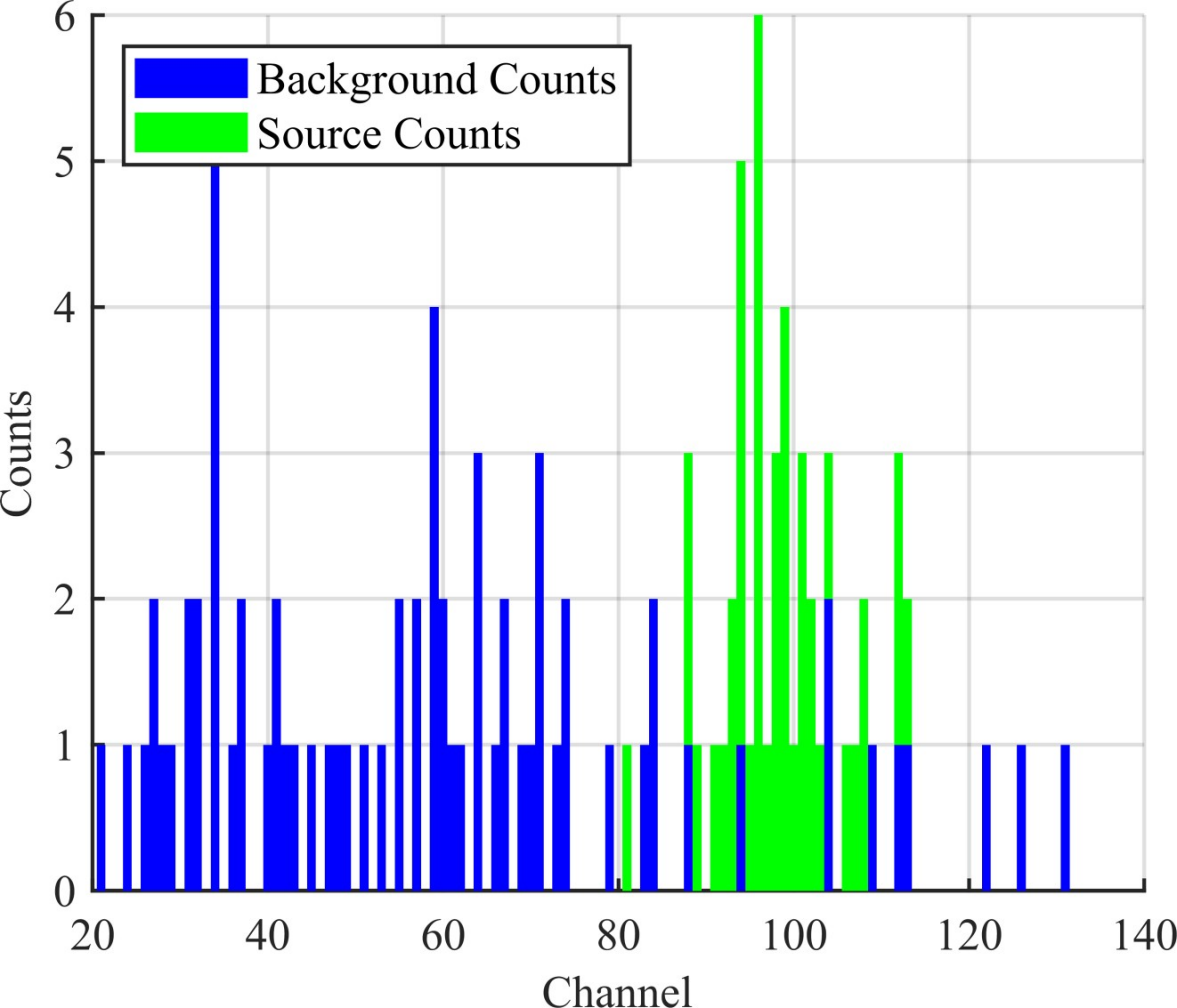
Simulated spectrum. One second of data with real background counts in blue and simulated source counts in green. Here, the visualized spectrum is showing the first 140 channels of 1024 channels–higher channels contain mostly zero counts and are not displayed.

For the MLE-Bayesian algorithm, the source and background counts need to be estimated (or separated). To accommodate this, a spectrum is generated for each collection instead of a gross count total. Background spectra were drawn from a large dataset collected on UIUC campus. The background spectra data was collected with a Kromek D3S gamma-ray detector on UIUC campus with no anomalous sources present. This data was provided from [18]. This background set consists of 14077, 1 s spectra with an average of 40±16 counts spread across 1024 detector channels (binned from the 4096 native to the D3S). The source spectra were synthesized via a statistical model as in [19]. This process first computes how many source counts are detected and then distributes them into a source spectrum for each collection. The number of source counts captured by the detector is computed as *c* = *ϵ*_*g*_ *Pois*(*I*/*d*^2^), with the source intensity *I*, the distance *d* meters between the detector and the source, and the geometric efficiency *ϵ*_*g*_–calculated from the face dimensions of the detector (0.5 in × 2.54 in for the D3S). Here, a random Poisson number was generated for each source spectrum synthesized and used to compute *c*, as indicated by *Pois*(*I*/*d*^2^). Then, for each count in *c*, a random Normal number was generated following *N*(*p*, *σ*^2^), where *σ* is defined by the FWHM of the detector and *p* is the peak channel (here, *p* = 100 for just overlapping the background and source spectra). This random Normal number indicates which channel that count is placed in. This synthesized source spectrum is computed and a background spectrum sampled for each measurement and are then combined for a total spectrum. An example of this spectrum with labelled counts can be seen in Fig 2.

### MLE-Bayesian localization scheme

The goal of both MLE and Bayesian inference is to extract properties of an unknown probability distribution. MLE provides a point estimate of distribution parameters while Bayesian inference yields a distribution for these parameters. In the context of source localization, we are interested in estimating the location ***r***∈(*x*,*y*) and intensity *I* of an unknown source given a series of measurements ***m***∈*m*_*i*_–formally, *P*(***r***,*I*|***m***). Bayesian inference updates an ongoing estimate (the posterior) of this via Bayes’ formula:

$$P{\left(r,I|m\right)}_{i}=\frac{P(m_{i}|r,I)\cdot P(r,I)}{P(m)}$$ (3)

where *P*(***r***,*I*) is the prior, the initial estimate of the distribution; *P*(***m***) is the probability of observing ***m***, which is typically computed via integration over the parameter space, though this is often intractable and involves more advanced computation techniques [2]; and *P*(*m*_*i*_|***r***,*I*) is the probability of most recent observation given ***r*** and *I*, constructed by substituting Eq 2 into Eq 1. For updating an ongoing estimate, the prior in Eq 3 can be substituted with *P*(***r***,*I*|***m***)_*i*−1_.

In MLE many observations are taken into account at once. Similarly, Bayesian schemes can involve multiple observations at each update step. In such cases, with *N* observations, *P*(*m*_*i*_|***r***,*I*) is recast as a likelihood function:

$$L(r,I)=\prod_{N}^{n}P(m_{n}|r,I)=\prod_{N}^{n}\frac{e^{-\mu_{n}}\mu_{n}^{m_{n}}}{m_{n}!}$$ (4)

where we have adopted the convenient notation of Miller et al. in the estimated source response *μ* as [6]:

$$\mu_{n}(r,I)=b_{i}+I\cdot s_{n}(r)\cdot 1_{\left\{\mathrm{not}\;\mathrm{blocked}\right\}}$$ (5)

Here, *s*_*n*_(***r***) is the expected detected counts due to a unit-strength source at location ***r***. The *n* can refer to either a detector at the *n*^*th*^ position or the *n*^*th*^ detector in a network. The researchers in [6] go on to approximate this distribution as Gaussian with variance equal to the count rate (Eq 5) of the test source.

The goal of MLE is to find parameters ***r*** and *I* which maximize L(r,I) as:

$$\left(\hat{r},\hat{I})=Argmax[L(r,I|m)\right]$$ (6)

Maximizing Eq 4 is equivalent to maximizing the log of the likelihood function, which replaces the product in Eq 4 with a summation. This optimization step is typically non-trivial, and depending upon technique, may result in parameters describing a local maximum rather than the global maximum [10]. In this approach, like that in [6], the likelihood function in Eq 4 is updated after every measurement, bypassing the integration required for Eq 3. Because the environment is discretized into a grid, there are roughly 100 possible source locations (depending on wall presence), marginalizing the multidimensional likelihood functions into 100 intensity-dependent functions as:

$$L_{r}(I)=\prod_{t}P(x_{t}|r,I)$$ (7)

For each ***r*** (each potential source location), after each time step *t*, the product is updated by multiplication of the newest likelihood term *P*(*x*_*t*_|***r***,*I*).

Here, the likelihood *P*(*x*_*t*_|***r***,*I*) is probed at 100 possible source intensities ranging from 0 to 10000 cnts/s (about double of what can be encountered during training of the RL navigation algorithm). While this does introduce hyperparameters into the scheme, the purpose of this implementation is to test stopping conditions, and so this represents an adequately formulated solution similar to popular methods. After each new collection, the likelihood of the source being at any one location is computed by acquiring the maximum of $L_{r}(I)$ – which is 100 discrete points at each ***r*** and so is performed easily. This likelihood map is then normalized so the sum is equal to 1. The initial distribution is set to uniform at all source locations and potential intensities.

Thus, a Bayesian-like approach is used to update an ongoing likelihood map by maximizing individual likelihood functions. In Miller et al. the background *b* in Eq 5 is assumed to be constant after recording a few seconds of data taken to be only background. We utilize the anomaly detection algorithm proposed in [19] to first detect source presence and then to compute estimated background and source counts after each step. This selection is a natural choice for background estimation as this anomaly detection scheme is designed for time-series, sparse gamma-ray data. The spectral background data outlined previously were used here.

For evaluating stopping conditions, the RL-guided search would terminate after a location achieved a likelihood of 0.9 or higher; this location serves as the source location estimate. In addition, distance requirements were tested to investigate if whether enforcing close-approach to high-likelihood locations would influence localization accuracy–i.e., the detector must be within a certain radius of the estimated location in order to terminate. For each trial, when the 0.9 likelihood threshold is crossed and for every step where it remains crossed, the distance between that location and the detector-agent location was computed. If this distance is the closest-approach so far, it is record as satisfying all distance requirement equal to or greater than that distance–e.g., a 5 m closest approach will satisfy a 5 m, 6 m, and 7 m (and so on) closest-approach requirement if those greater distances qualified yet due to sub-threshold likelihood values. The navigation was permitted to run until it satisfied a 0 m distance requirement, noting the location accuracy and steps for every distance along the way. For testing this approach, set initializations were used: the detector agent was initialized to grid point [1, 10] (the upper-left corner), the source was initialized to grid point [2, 8] (near the bottom-right corner), and source intensity *I* was set to 2000 cnts/s. This intensity equates to roughly 18 cnts/s being detected at the initialization location. The number of steps taken to produce a localization estimate and its accuracy were recorded and 50 trials were conducted. With this evaluation, no walls were generated, as this methodology cannot handle the attenuation complexity structures would introduce. Hite and Mattingly [2] have proposed a methodology for tackling this, but it relies upon a detector-network and would likely push computation times orders of magnitude larger than the 1 s dwell times.

### Q-Leaning convolutional neural network for navigation

The convolutional neural network (CNN) for source localization and navigation used in this study is taken from [14], and so an overview of the approach will be given here. The problem at hand is navigating a detector agent within the simulated environment to the source location. This problem is formulated as a finite discrete Markov decision process where the agent responds to a sequence of states (*s*) with one of four discrete actions (*a*) in anticipation of a reward (*R*). The action space consists of moving up, down, left, or right (from a top-down view). The state space consists of three matrices describing the number of measurements taken at each grid point, the mean of all measurements taken at each grid point, and a map of the environment. During training, the action policy is learned based upon compassion between the network output and the computable reward via the Q function. The training reward for each time step is:

$$R_{t}=\{\begin{array}{c} 0.5,\;\mathrm{if}\;\mathrm{the}\;\mathrm{agent}\;\mathrm{moves}\;\mathrm{closer}\;\mathrm{to}\;\mathrm{the}\;\mathrm{source}\\ -1.5,\;\mathrm{otherwise} \end{array}$$ (8)

This reward structure is deliberately asymmetric to encourage localizing as quickly as possible. The goal of Q learning within RL is to find the optimal action value function Q*(*s*,*a*):

$$Q^{*}(s,a)=\underset{\pi }{\max}\;E[\sum_{t^{\prime }=0}\gamma^{t^{\prime }}R_{t^{\prime }}|s,a,\pi ]$$ (9)

**Fig 3:**
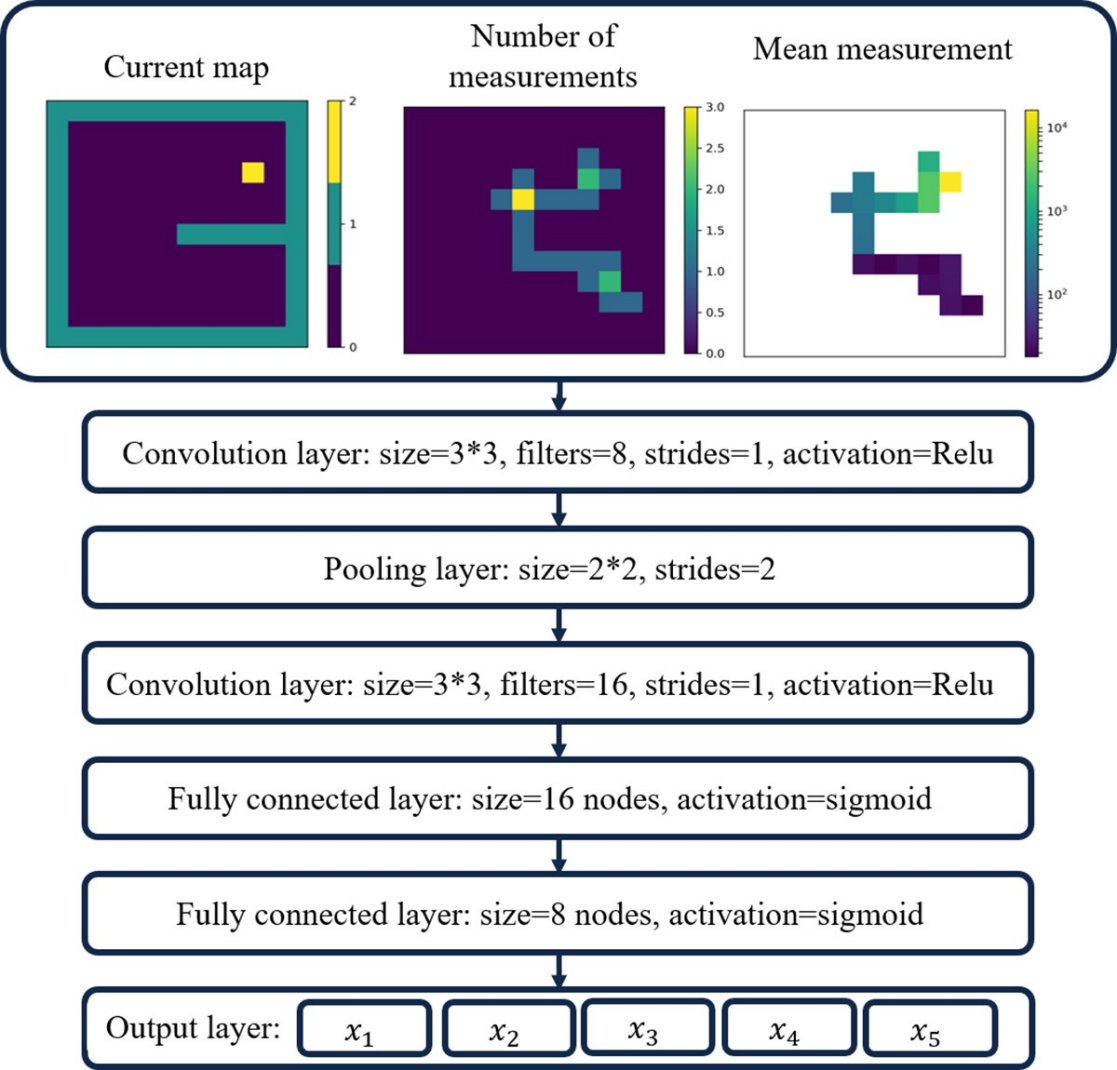
CNN with stopping architecture. Architecture of the CNN with self-stopping. The only difference between this self-stopping network and the navigation only network is the addition of a fifth output representing a termination action. Removing *x*_5_ would yield the network architecture used in [14].

This function represents the maximum expected cumulative future reward beginning from state *s*, with action *a*, action policy *π*, discounted by *γ*, and accumulated for future steps *t*′. A CNN, whose inputs are the state matrices (environment and history) and whose output is the expected cumulative reward for taking each action (the greatest of which is taken to be the action in testing), is trained to approximate this Q function. The architecture of this network can be seen in Fig 3 if the fifth visualized output is dropped.

This network was trained over one million episodes, with source intensity varying uniformly between 3000 and 7000 cnts/s between episodes. Every 100 episodes, a checkpoint was created and 30 evaluation episodes were performed with fixed network parameters. For more specific training and implementation details, see [14]. The researchers provided the trained CNN produced from their work (https://github.com/rillab/RLRadiation).

### Q-Leaning convolutional neural network with self-stopping

Notably, the work in [14] lacks an independent stopping condition, in which the agent terminates its search. Here, we propose an expansion of this work to include a fifth action into the action space: a stopping condition. This addition is a natural extension of the CNN and results in a small architectural change, see Fig 3.

With the new action space, the reward structure needs to be amended. If the agent takes a move action (up, down, left, or right), then the reward definition in Eq 8 is used. If the agent takes a stop action, then the reward is computed as:

R=−1.5⋅{minimumstepstosourcelocation} (10)

The minimum number of steps to reach the source location already needs to be computed for calculating the future reward in the original network, and so this adds no computational complexity. Keep in mind, reward is only computed during training, when the source location is known to the training algorithm but not the CNN itself. With this reward structure, the further the agent is from the source when it stops, the more it is punished. Using -1.5 as opposed to -0.5 prioritizes getting as close to the source as possible over taking fewer steps. The same training regimen and parameters as in [14] were used.

For evaluating localization accuracy, 1000 episodes were run with the trained network with identical testing initialization as to the MLE-Bayesian scheme–taking note of localization speed and accuracy. In addition to this, 1000 episodes with randomly generated walls were also run. Each episode ended when either 100 steps were taken or the stopping action was taken. The trained CNN produced from this work can be found at https://github.com/rillab/.

## Results

First presented are the performance details of the MLE-Bayesian scheme and the training results of the CNN with stopping in respective subsections. Then, each algorithm’s localization results are presented in a comparison subsection.

### MLE-Bayesian localization performance

**Fig 4:**
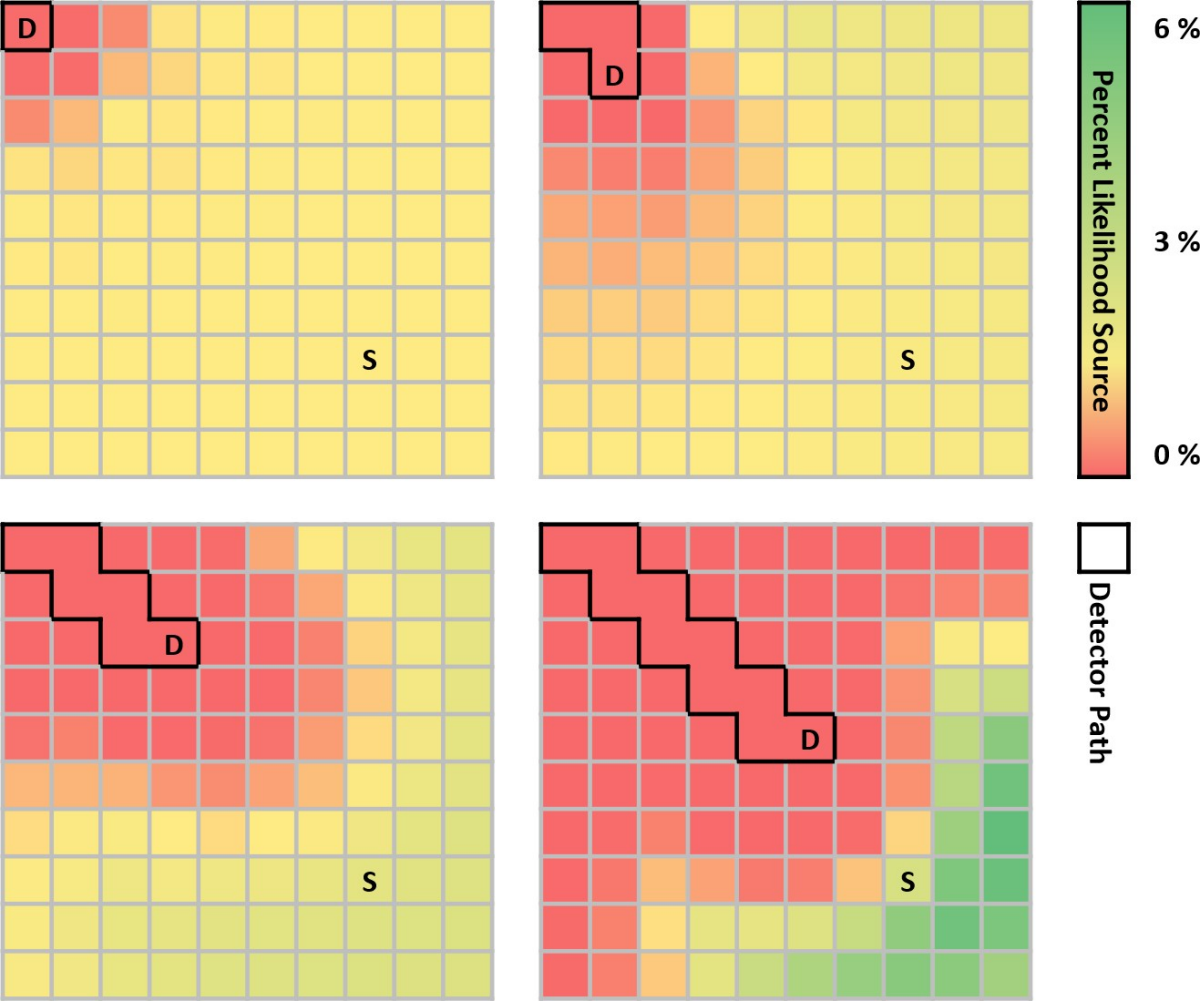
MLE-Bayesian updating scheme. Presented is a demonstration of the MLE-Bayesian approach as it localizes a source of intensity 2000 cnts/s. Shown here are steps 1, 3, 6, and 10 taken with the trained CNN. The detector-agent is indicated with the “D”, its path with the black borders, and the source with the “S”.

The MLE-Bayesian localization scheme was guided by a CNN for navigation, separating source location estimation and the decision-making process for choosing the next surveying location. After each step, a likelihood map of source location was updated–this process is illustrated in Fig 4, showing the likelihood map after steps 1, 3, 6, and 10. The environment was initialized as described in the Methods section with source intensity *I* set to 2000 cnts/s. For this demonstration, the source and background count estimation algorithm was not used.

**Fig 5:**
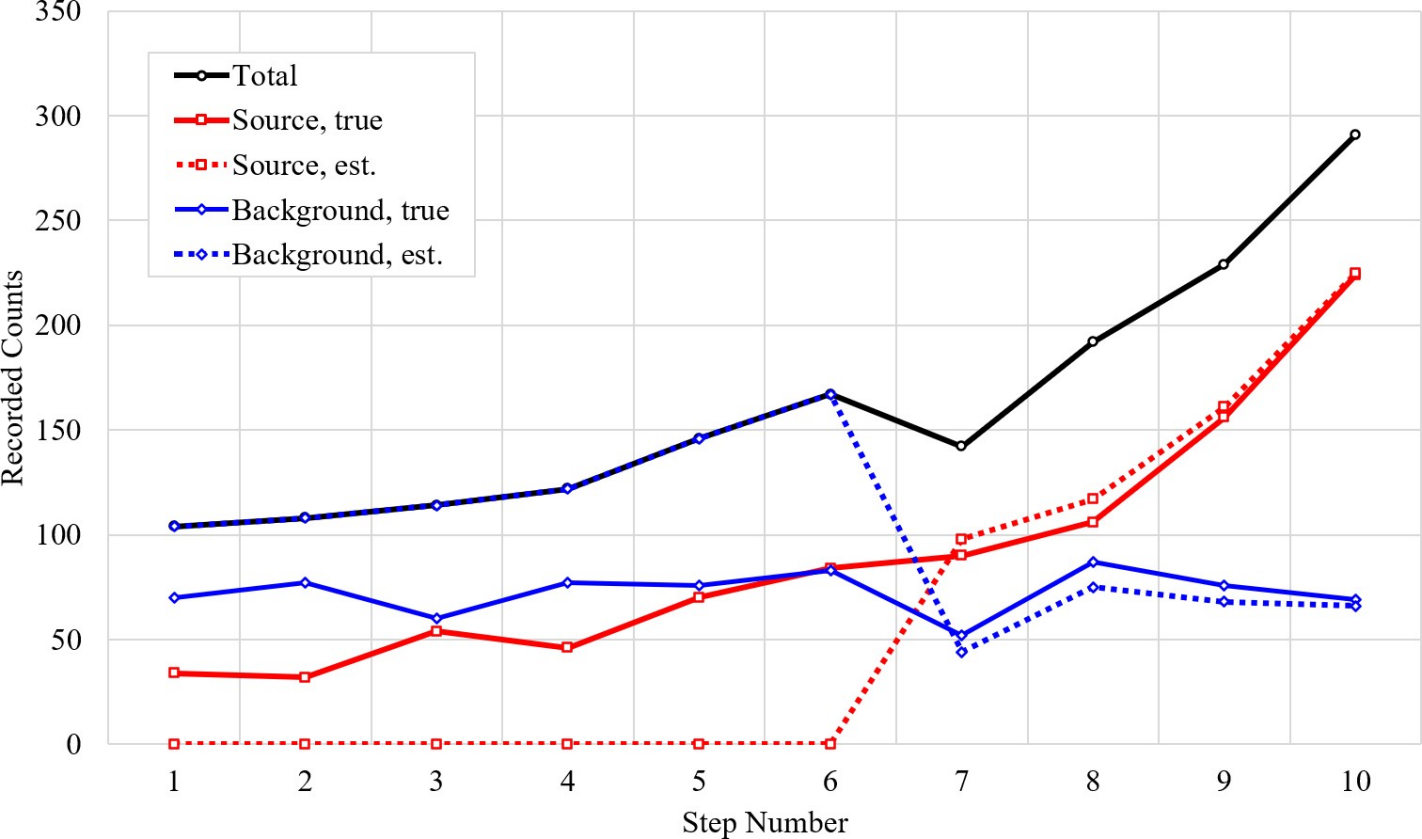
Example of count prediction. Representation of the counts and their quality as seen by the MLE-Bayesian approach with trained CNN. An anomaly is detected after the 6^th^ collection, leading to a good estimated separation of source and background counts. Background counts are shown in blue with diamonds–true is solid while estimated is dashed. Source counts are shown in red with squares–true is solid while estimated is dashed. Total counts is in black with circles.

The performance of a trial with count estimation is presented in Fig 5 with environmental parameters the same as above. An anomaly was detected after step 6, and source and background counts were separated thereafter. With the environmental initialization described and over 50 trials, an anomaly was detected after a median of six steps with a lower quartile of five steps and an upper quartile of seven steps. After an anomaly was detected, estimated and true source counts had an average correlation coefficient of 0.997 and the estimated and true background counts had an average correlation coefficient of 0.72. This demonstrates the MLE-Bayesian scheme is using high quality information during its estimation process.

### CNN with self-stopping training results

**Fig 6:**
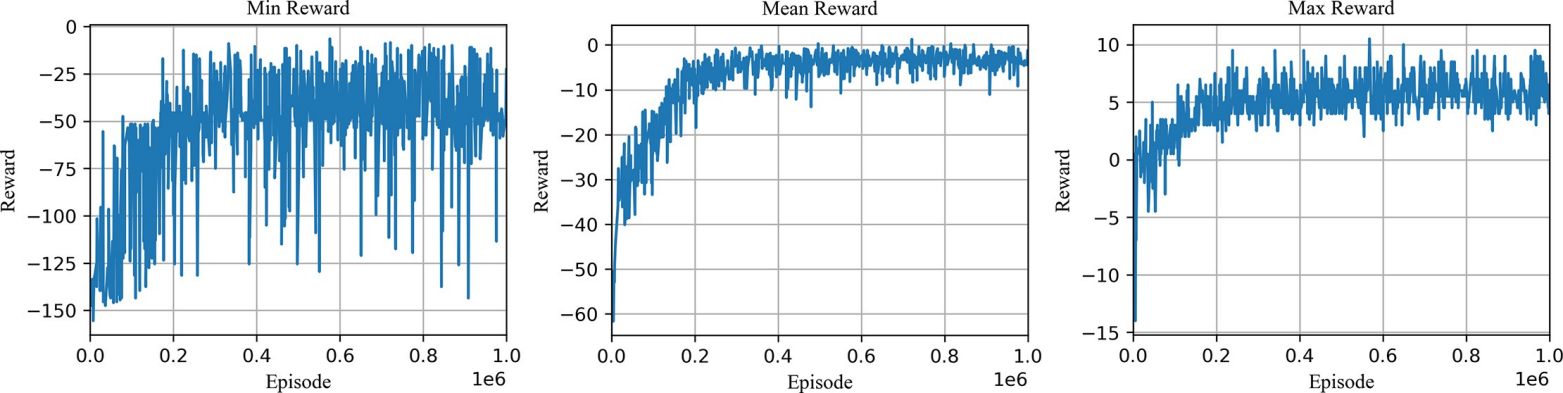
Validation episode rewards for CNN with self-stopping during training. Shown is the (A) minimum, (B) mean, and (C) maximum rewards for the 30 validation episodes performed every 100 training episodes with network parameters held constant.

The CNN with stopping was trained over the course of one million episodes with each episode terminating after either 100 steps were taken or the stopping action was taken. Every 100 episodes, a checkpoint of model parameters and performance was created. Along with this, a validation test was run with 30 randomly initialized episodes and fixed network parameters, yielding mean, minimum, and maximum rewards for each validation set–as seen in Fig 6.

The reward is a performance metric for both navigation and stopping. Specifically, the reward from navigation is incrementally given after each step in each localization trial while the reward from stopping occurs once per trial; the raw reward is a combination of these two factors. So, while the overall performance of the algorithm can be monitored during training via the reward curves, the navigation and stopping performance must be individually tested (following sections). Despite this, observing increasing reward is a promising sign during training. The mean test reward converges to near-zero after about 700k episodes. The minimum and maximum test rewards were much more varied than the mean test reward, converging to -40 and 5, respectively. This behavior can be explained with outlier scenarios the network either did not adequately handle or encountered regularly. The 842,500^th^ checkpoint was selected for further testing as it yielded the greatest minimum test reward.

### Localization results

Localization of a radioactive source was measured on two criteria: localization error (meters between estimated and true source location) and localization time (number of steps taken). This dual metric is necessary for consideration due to the task at hand: exceptional localization speed with poor localization accuracy would make for a poor algorithm, and vice versa.

**Fig 7:**
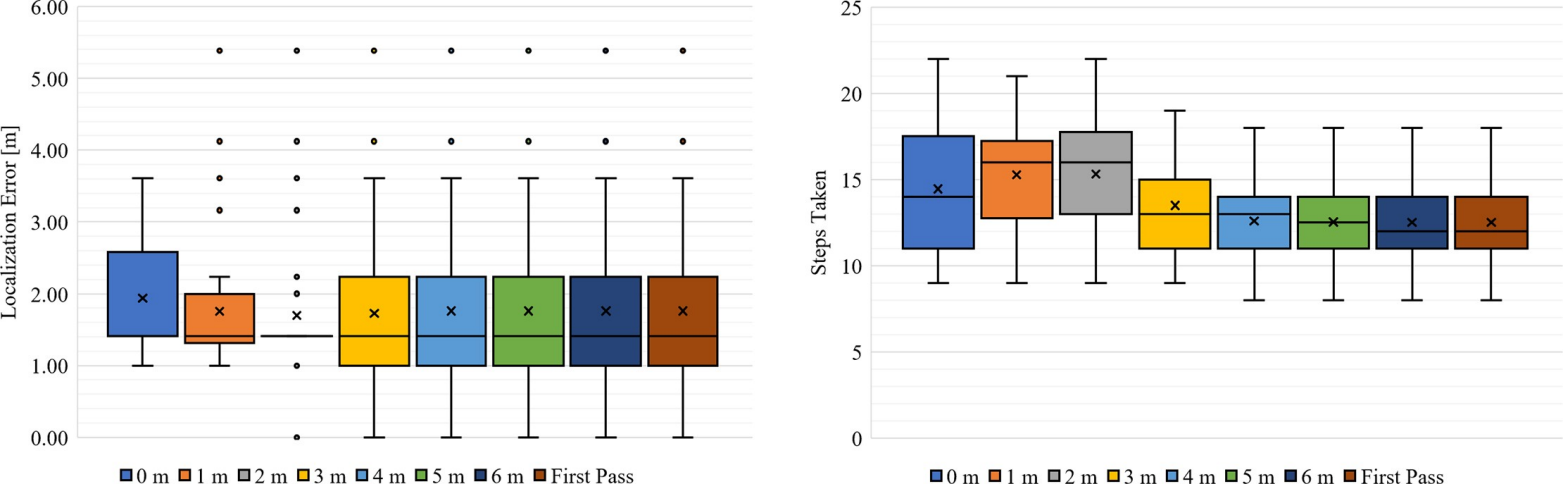
Localization error and steps taken for MLE-Bayesian approach. Whisker and box plots showing (A) the distribution of localization error and (B) steps taken to localize by the MLE-Bayesian method. The “x” marks the mean of the distribution.

Over the 50 trials for the MLE-Bayesian test, the median localization error was 1.41 m for all distance requirements (representing a diagonal displacement), as seen in Fig 7A. The localization accuracy with no distance requirement is labelled “First Pass,” indicating it is simply the first time the likelihood threshold is passed. Each other distance requirement represents the performance of the system if the detector had to move within that distance of the 0.9 likelihood location to terminate. No trial satisfied a distance requirement of 6 m without also satisfying a smaller distance requirement, and so all these distributions match the 6 m case, as seen with the “First Pass” case with essentially has an infinite distance requirement. It should be noted that the localization error is discretized in a sense due to the grided environment, making only certain errors achievable. While the 1 m distance requirement yielded the narrowest range of errors, this requirement appears to have little effect on accuracy performance. The steps taken was more noticeably affected by the distance requirement (due to the need to move close to the estimated source), but still had relatively consistent performance, seen in Fig 7B.

**Fig 8:**
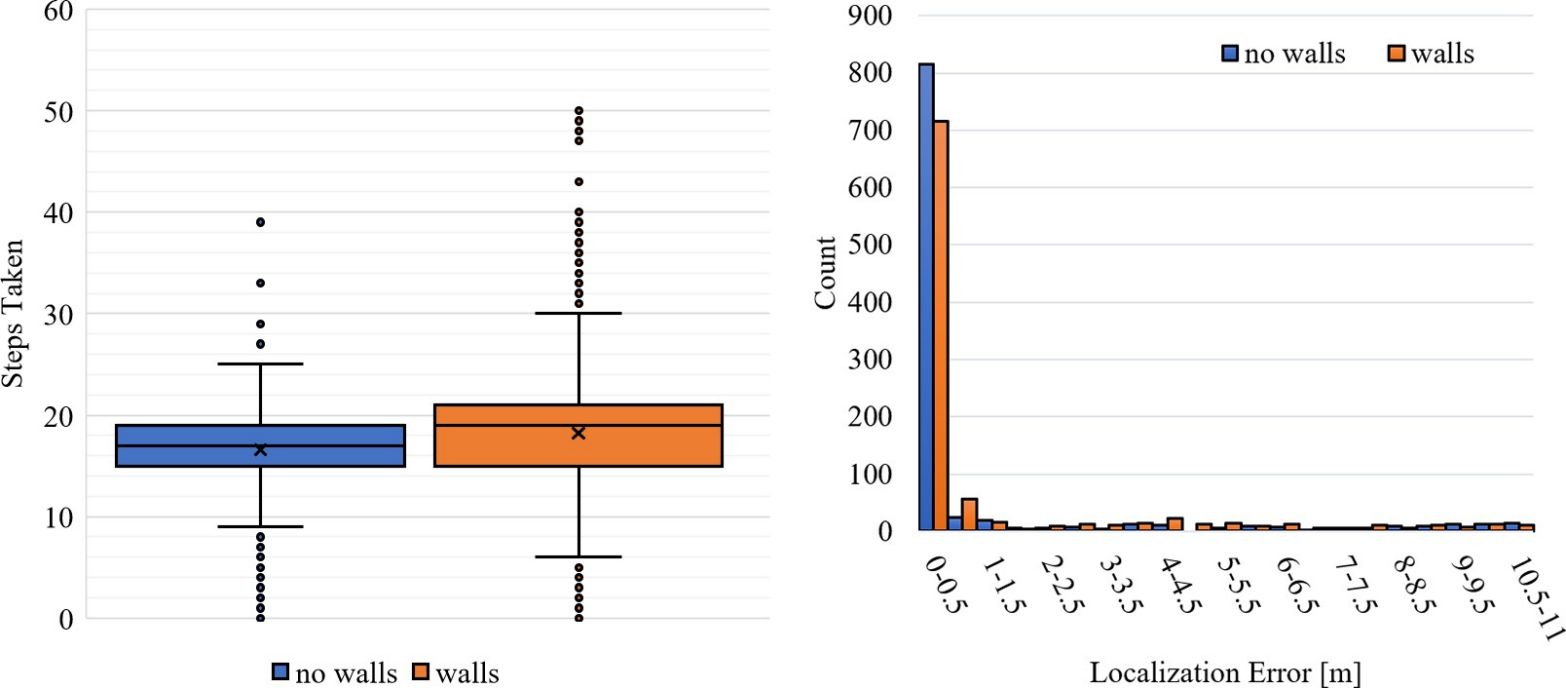
Localization error and steps taken for CNN with self-stopping. (A) Histogram of localization error and (B) whisker and box plots of the steps taken to localize by CNN with self-stopping. The “x” marks the mean of the distribution.

Over the 1000 trials for either case, the CNN had a median error of zero whether walls were present or not. Further, the addition of walls had only a slightly negative effect on localization error distribution, visible in Fig 8A. The CNN speed performance was on par with the slower MLE-Bayesian distance requirements, with a median of 17 and 19 steps for no wall and with walls, respectively. Similar to localization accuracy, wall presence did have a slightly negative effect on the spread of steps taken, seen in Fig 8B.

## Discussion

Disregarding the trials with walls for a moment (as they were exclusive to the CNN with stopping), a trade-off is evident between the two investigated approaches: that of speed and accuracy. The MLE-Bayesian approach tended to localize the source slightly faster, while the CNN approach localized much more accurately. With the nature of the environment and simplicity of the simulation, the comparatively worse localization performance of the MLE-Bayesian approach may be due to the strict threshold set for stopping. Recall that a given grid-point needed a likelihood equal to or exceeding 0.9 for the search to terminate. Because of the randomness of radiation emission from the source and background–and despite the excellent separation of source and background counts–, requiring such high confidence may hinder localization accuracy in some situations. Selecting the optimal threshold for this scenario may be inadequate for others where source or background strength is drastically different. This hyperparameter is ultimately a user-defined variable and selecting different values may yield different localization results. The speed of the MLE-Bayesian approach may also increase with lower threshold values because less evidence (collections/steps) is then required. Lowering this parameter, however, may cause a premature termination, stopping the search before the agent has navigated closer to the source where it would have access to more information. The distance requirement had little effect on performance, but such a criterion may be required or desired for larger scale searches.

The CNN with self-stopping had excellent performance for localization despite no separation of source and background counts, a process which could easily be incorporated into the scheme. The speed of its localization is 5 to 7 steps slower than the MLE-Bayesian approach, but it is required to navigate to the exact estimated source location while the MLE-Bayesian approach may stop further away. The strength of this approach is two-fold: 1) no hyperparameters need to be determined outside of training, and 2) exposure to novel environments during training can expand its applicability. While this model was trained with a range of source intensities and uniform background strength, incorporating a range of background strengths during training comes with only the trade-off of more training. Further, this network is able to navigate around obstacles, implicitly accounting for attenuation of source. The same cannot be said for the MLE-Bayesian approach which would need to explicitly account for this. While possible, adding such physics computations would likely increase the dwell time beyond one second and also reduce the confidence in estimates due to potential error in estimated attenuation coefficients.

Generally, the CNN with self-stopping approach is more flexible and accurate than the MLE-Bayesian approach while requiring more steps. The ML approach comes with a heavy upfront cost in training but can account for many variables that would otherwise need to be accounted for during deployment of a statistical model.

## Conclusion

This study compares two general approaches for terminating an ML-guided source search: one in the ML field of RL and the other using an MLE and Bayesian statistical strategy. As small drone technology becomes more viable for national security efforts–specifically in source localization tasks, it is necessary to investigate single-detector source localization strategies and all the hurdles in their implementation. Early efforts using ML for this have demonstrated success in quick and accurate localization and in the handling of navigational obstacles. These methods did not, however, sufficiently incorporate a method for stopping the search.

Comparing an MLE-Bayesian based approach and a reinforcement learning CNN with self-stopping approach for ending a search, the pure ML scheme localized with much greater accuracy but did so in a greater number of steps. The network that contained a stopping action had a median localization of accuracy of 0 m while the mixed ML-Bayesian approach only attained a best performance median accuracy of ~1.5 m. This increase is significant while considering the 10 m × 10 m environment. The speed of localization did diminish when using the stopping action network, however, lagging to an 17 step median over the 12 step median of the mixed MLE-Bayesian approach. Further, the benefit of the MLE-Bayesian approach is it provides a likelihood distribution of source presence at every area of interest and is based upon radiation physics. However, the drawbacks are that: 1) hyperparameters corresponding to confidence and source strength are required, and 2) accounting for structures in the search area for attenuation is challenging and may increase localization time. The CNN with stopping requires considerable upfront training, but because the drone is guided by ML, such training is already necessary. With ML, the generalizability is dependent upon the diversity and rigor of training, leading to potentially unaccounted for circumstances. For localization approaches depending on ML for navigation, however, this problem already needs to be addressed. As such, adding a stopping action is recommended.
